# Anti-transcription intermediary factor 1 gamma (TIF1γ) antibody-positive dermatomyositis associated with ascending colon cancer: a case report and review of the literature 

**DOI:** 10.1186/s13256-021-02664-1

**Published:** 2021-03-22

**Authors:** Ryohei Ono, Tomohiro Kumagae, Mari Igasaki, Takaaki Murata, Masaki Yoshizawa, Izumi Kitagawa

**Affiliations:** 1 Department of General Internal Medicine, Shonan Kamakura General Hospital, 1370-1 Okamoto, Kamakura, Kanagawa 247-8533 Japan; 2 Department of Surgery, Shonan Kamakura General Hospital, 1370-1 Okamoto, Kamakura, Kanagawa 247-8533 Japan; 3 Department of Rheumatology, Shonan Kamakura General Hospital, 1370-1 Okamoto, Kamakura, Kanagawa 247-8533 Japan

**Keywords:** Dermatomyositis, Anti-transcription intermediary factor 1 gamma, Anti-TIF1γ antibody, Cancer, Malignancy

## Abstract

**Background:**

Anti-transcriptional intermediary factor 1 gamma (TIF1γ) antibody is a marker for predicting cancer association in patients with dermatomyositis (DM). The overall survival rate in DM patients with cancer was reported to be considerably worse than that in DM patients without cancer. However, the treatment for cancer-associated DM remains controversial, because the treatment priority between surgical resection for the tumor and internal treatments, including glucocorticoids, immunosuppressive agents, and intravenous immune globulin, has not been established.

**Case presentation:**

We report the case of a 57-year-old Japanese man diagnosed with anti-TIF1γ antibody-positive DM associated with ascending colon cancer. His clinical symptoms included facial and brachial edema, muscle weakness, dysphagia, myalgia, and rash. Physical examination revealed periorbital edema and Gottron's papules over his knuckles with brachial edema, and tenderness and weakness of the proximal limb muscles. The findings of hyperintense muscles in T2-weighted sequences of brachial contrast-enhanced magnetic resonance imaging and the infiltration of lymphocytic cells and CD4-positive lymphocytes from muscle biopsy were compatible with the diagnostic criteria for dermatomyositis. Anti-TIF1γ antibody was positive by immunoprecipitation assay. He first started internal treatment including intravenous immunoglobulin, steroid pulse, prednisolone, and azathioprine, followed by surgical resection for the tumor because of the elevation of creatine kinase and progression of dysphagia. However, clinical symptoms did not improve, and the patient died 6 months later.

**Conclusions:**

We faced difficulties in determining the treatment priority between surgical resection and internal treatment for our case; therefore, this case would be educational for readers. We searched PubMed to identify English-language case reports of anti-TIF1γ antibody-positive dermatomyositis with malignancy and found 21 reported cases. We herein review and summarize previously reported cases of anti-TIF1γ antibody-positive DM with malignancy. Cancer screening is essential in patients with anti-TIF1γ antibody-positive dermatomyositis because it is associated with a high prevalence of malignancies. Our review revealed that initial surgical treatment should be recommended for better prognosis if the general condition allows.

## Background

Dermatomyositis (DM) is an inflammatory myopathy characterized by skin rash and progressive, symmetrical weakness of the proximal muscles [1, 2]. DM has been shown to be associated with malignant disease [3]. The overall survival rate in DM patients with cancer was found to be considerably worse than that in DM patients without cancer [4]. Recently, an anti-transcriptional intermediary factor 1 gamma (TIF1γ) antibody was reported as a marker for predicting cancer association in patients with DM, since TIF1γ, which regulates the tumor growth factor pathway, has been reported to be associated with tumor growth in some malignancies [5]. In a meta-analysis, Trallero-Araguas *et al.* reported that the pooled sensitivity of anti-TIF1γ antibody for diagnosing cancer-associated DM was 78%, whereas specificity was 89% [6]. The treatment for cancer-associated DM remains controversial, because the treatment priority between surgical resection for the tumor and internal treatments, including glucocorticoids, immunosuppressive agents, and intravenous immune globulin, has not been established. We searched PubMed to identify English-language case reports of anti-TIF1γ antibody-positive dermatomyositis with malignancy and found 21 reported cases [7–27]. Herein, we report a case of anti-TIF1γ antibody-positive dermatomyositis associated with ascending colon cancer; previously reported cases of anti-TIF1γ antibody-positive dermatomyositis with malignancy are reviewed and summarized. This case may provide a unique perspective for readers and illustrate the difficulties in determining treatment priority between surgical resection and internal treatment.

## Case presentation

A 57-year-old Japanese man presented with a 1-month history of progressive symptoms of facial and brachial edema, muscle weakness, dysphagia, myalgia, and a symmetrical widespread rash on his limbs and hands. He denied recent common cold symptoms. He was also noted to have unintentional weight loss (3 kg over 1 month). His medical and family histories were unremarkable. He was diagnosed with type 2 diabetes mellitus 8 years ago, but he did not go to the hospital until this visit.

**Fig. 1:**
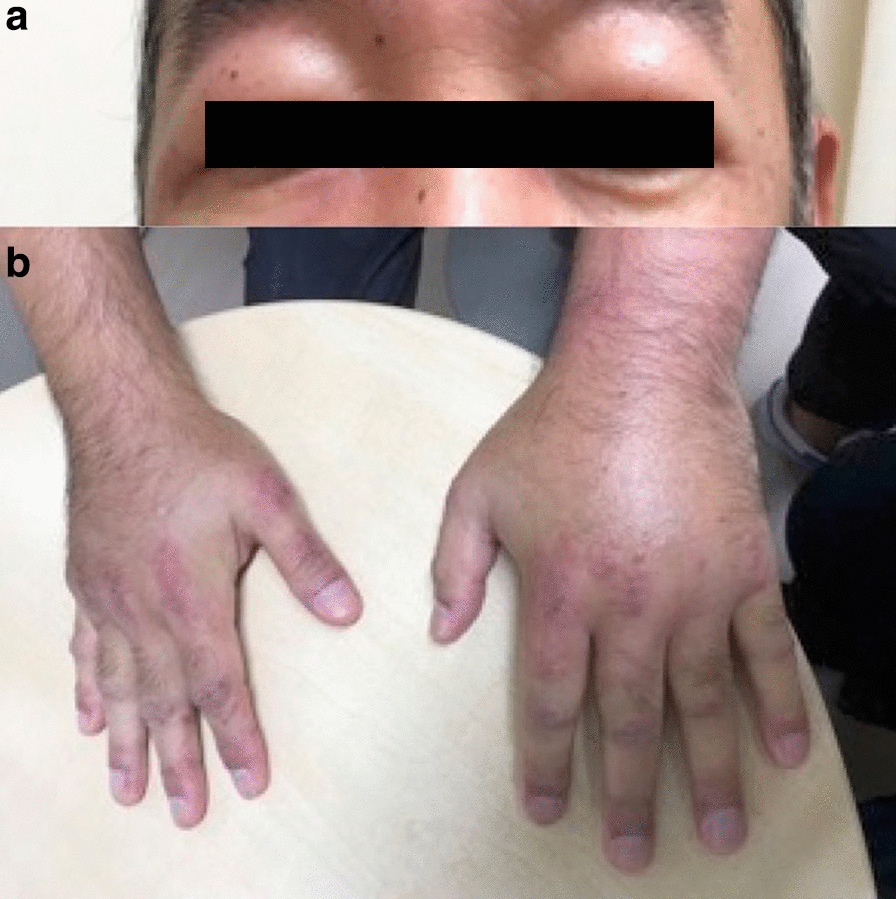
Physical examination revealed periorbital edema (**a**) and Gottron's papules over his knuckles (**b**)

Vital signs showed that the patient was afebrile, with a heart rate of 90 beats per minute, blood pressure of 120/78 mmHg, normal respiratory rate, and oxygen saturation of 99% on room air. Physical examination revealed periorbital edema (Fig. 1a) and Gottron's papules over his knuckles (Fig. 1b) with brachial edema, and tenderness and weakness of the proximal limb muscles. Laboratory evaluation revealed elevated levels of creatine kinase (5002 U/L; reference range 30–175 U/L), aspartate transaminase (120 U/L; reference range, 12–35 U/L), alanine aminotransferase (46 U/L; reference range 6–40 U/L), lactate dehydrogenase (440 U/L; reference range 119–229 U/L), D-dimer (9.1 μg/mL; reference range <1.0 μg/mL), and hemoglobin A1c (9.2%; reference range 4.6−6.2 %); however, white blood count, C-reactive protein, hemoglobin, electrolytes, lipid profile, and renal function were normal. Hepatitis B and C, and HIV serologies were all negative. Chest radiography showed no consolidation. Respiratory function tests, electrocardiogram, and echocardiogram were unremarkable. Because of the history and significantly elevated muscle injury biomarkers, we suspected inflammatory myositis. The patient underwent further evaluation to investigate the probable diagnosis.

**Fig. 2:**
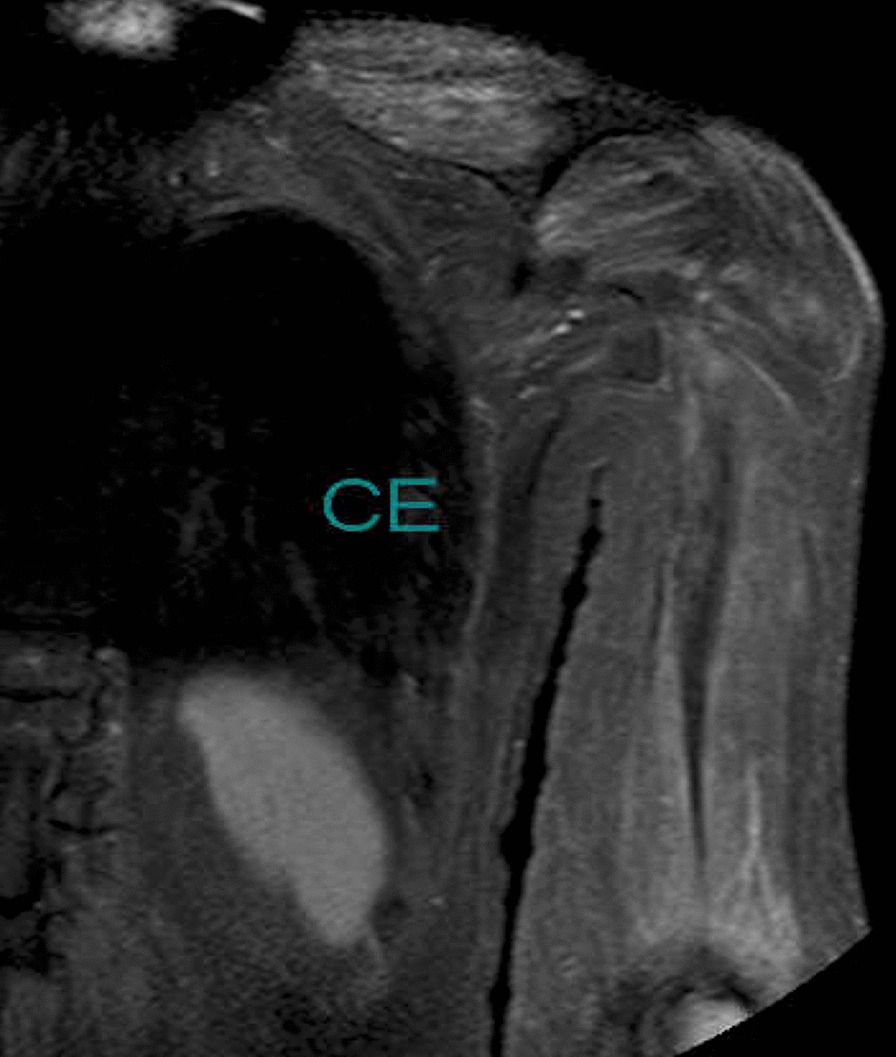
Brachial contrast-enhanced magnetic resonance imaging demonstrated hyperintense muscles in T2-weighted sequences

**Fig. 3:**
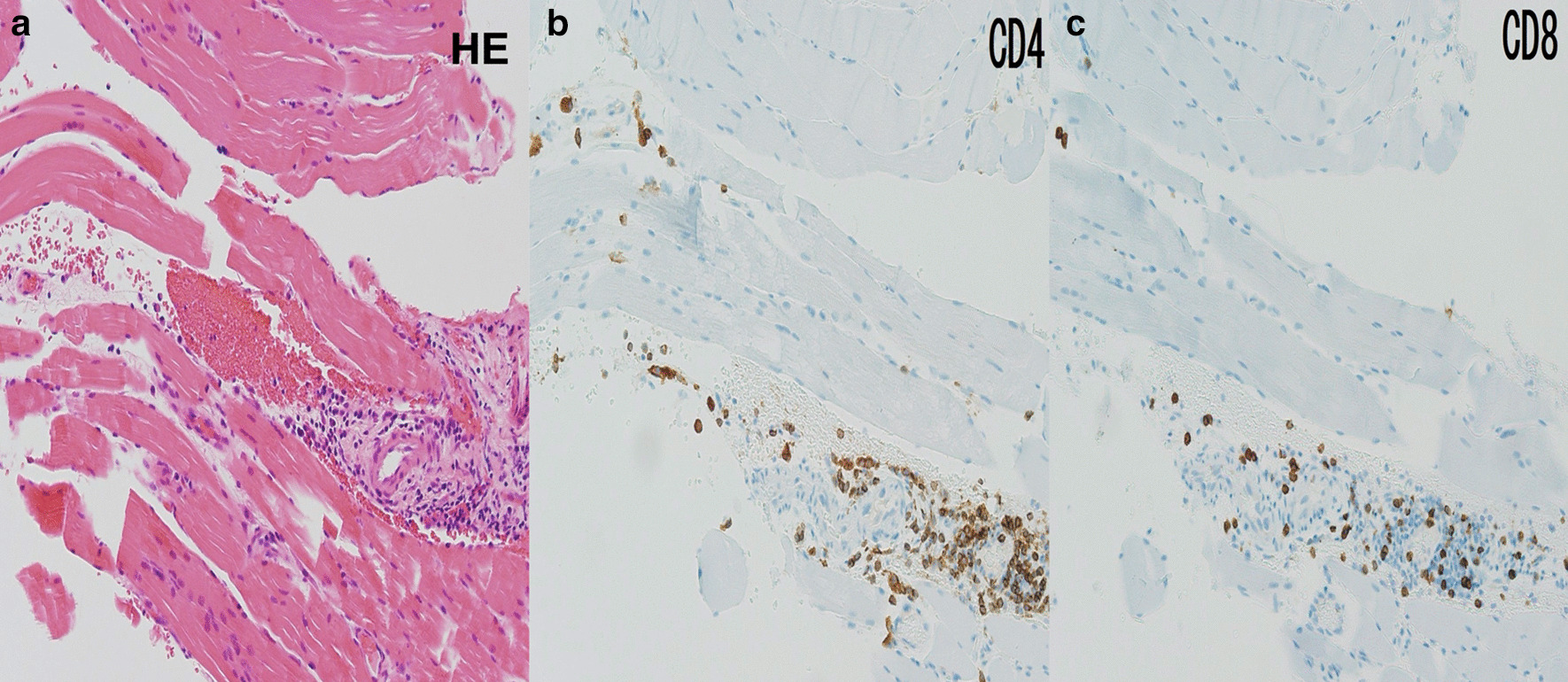
**a** Hematoxylin and eosin stain (×40 magnification) of the muscle showing the infiltration of lymphocytic cells. **b** CD4 antibody staining (×100 magnification) and **c** CD8 antibody staining (×100 magnification) confirmed the predominant presence of CD4-positive lymphocytes

**Fig. 4:**
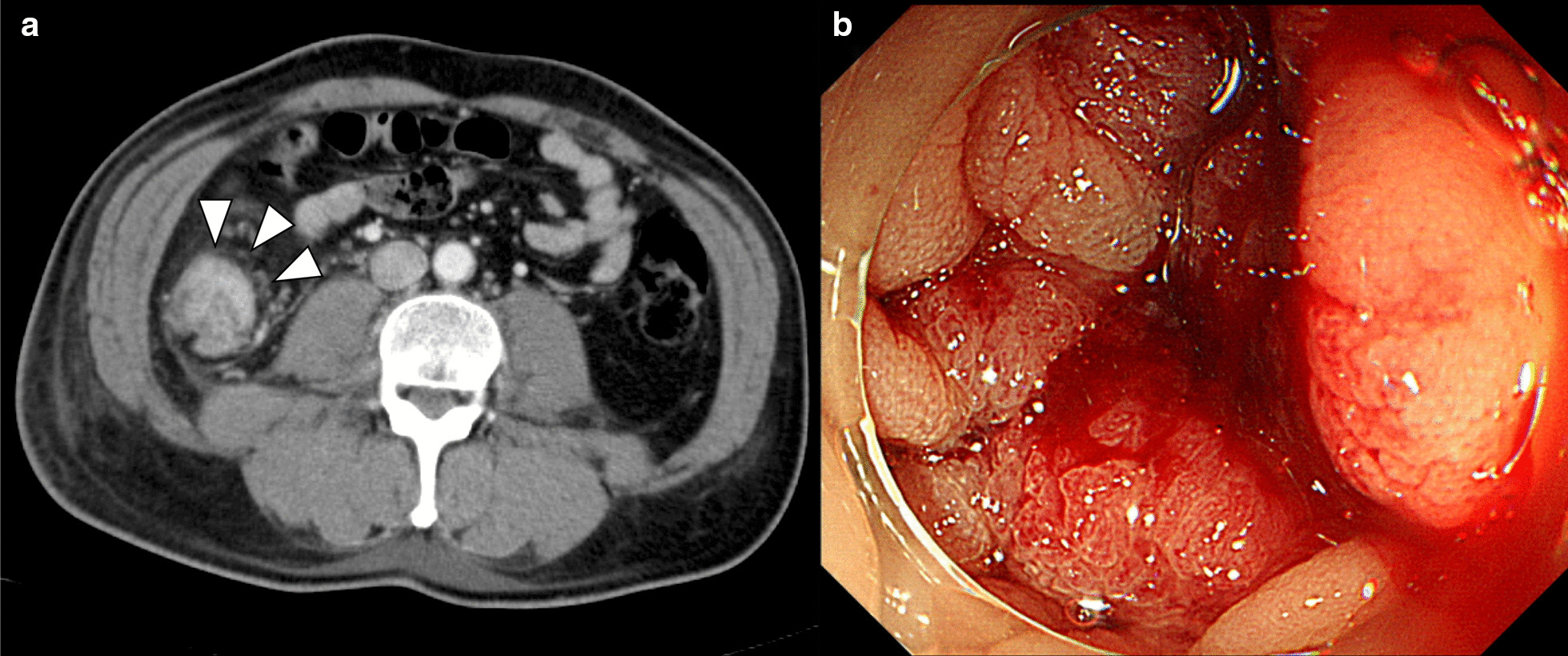
Colonoscopy revealing the ascending colon tumor. Contrast computed tomography showing a tumor mass in the ascending colon (white arrows) with no other notable metastases (**a**), and colonoscopy revealing an ascending colon tumor (**b**)

**Fig. 5:**
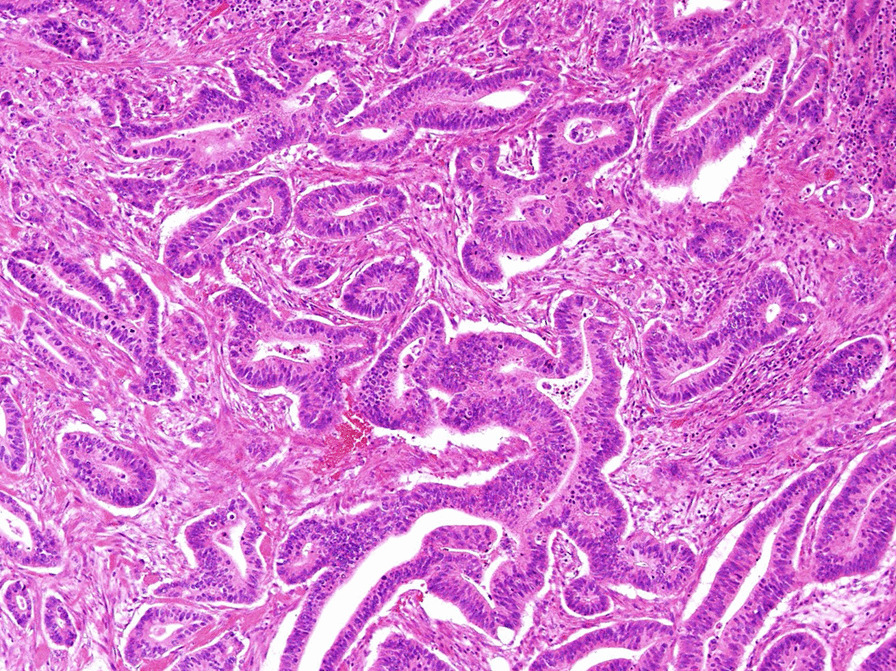
The histopathological findings of the biopsy from the ascending colon showing well-differentiated tubular adenocarcinoma (hematoxylin and eosin stain, ×400 magnification)

Additional laboratory data demonstrated that antinuclear antibody was positive at 1:40 with a speckled pattern. In addition, anti-TIF1γ antibody was positive by immunoprecipitation assay, although other markers including anti-aminoacyl-tRNA synthetase, anti-melanoma differentiation-associated gene 5 antibody, and anti-Mi2 antibody were negative. Brachial contrast-enhanced magnetic resonance imaging (MRI) demonstrated hyperintense muscles in T2-weighted sequences (Fig. 2). A biopsy from the biceps brachii muscle was performed, and the infiltration of lymphocytic cells and CD4-positive lymphocytes was confirmed (Fig. 3). These findings were compatible with dermatomyositis. Since anti-TIF1γ antibody has been associated with malignancies in dermatomyositis patients, we performed a whole contrast computed tomography scan and endoscopy. Contrast computed tomography showed a tumor mass in the ascending colon with no other notable metastases (Fig. 4a). Colonoscopy revealed an ascending colon tumor (Fig. 4b). The histopathological findings of the biopsy from the ascending colon showed well-differentiated tubular adenocarcinoma (Fig. 5). A diagnosis of anti-TIF1γ antibody-positive dermatomyositis with ascending colon cancer (cT4aN2M0, clinical stage IIIb) was made.

**Fig. 6.**
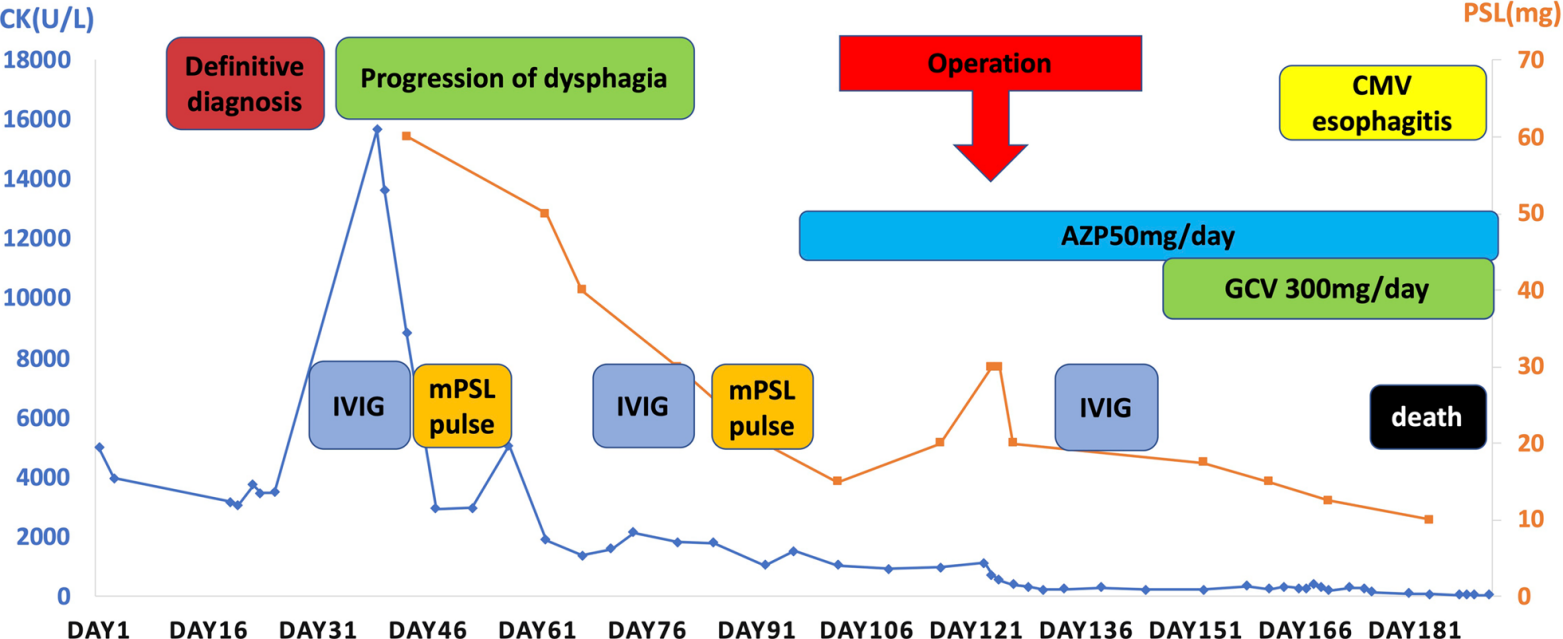
Clinical course. *AZP* azathioprine, *CMV* cytomegalovirus, *GCV* ganciclovir, *IVIG* intravenous immunoglobulin, *mPSL* methylprednisolone.

The clinical course is shown in Fig. 6. He was initially scheduled to undergo surgical resection for the ascending colon cancer after the definitive diagnosis; however, elevation of creatine kinase (15,667 U/L) and progression of dysphagia were noted before the operation. Thus, we decided that medical treatment should be performed first. Subsequently, intravenous immunoglobulin (IVIG) and 1 mg/kg of prednisolone with slow tapering of the dose was started from day 40, and the level of creatine kinase decreased significantly. However, his dysphagia did not improve, and creatine kinase was elevated again on day 57. Thus, 1 g/day of methylprednisolone (mPSL) for three consecutive days was administered from day 58. After the second cycle of IVIG (on day 75) and steroid pulse therapy (on day 81), 50 mg/day of azathioprine was started on day 97 because creatine kinase was decreased but the dysphagia persisted. As the patient’s condition had deteriorated, manual muscle testing of his limb was grade 2 out of 5, and medical treatment was considered ineffective, PSL was increased from 15 to 20 mg per day and surgery including right hemicolectomy, gastrostomy, and tracheostomy was performed on day 124.

**Fig. 7:**
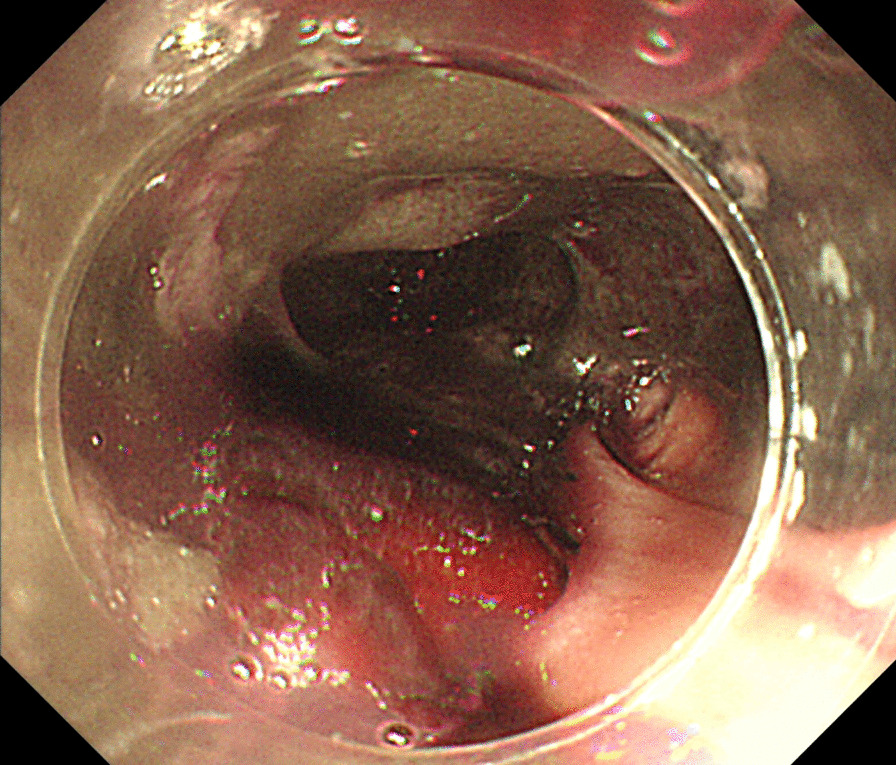
Gastroscopy showing cytomegalovirus esophagitis

After surgery, the patient was almost bedridden due to disuse syndrome despite continuous rehabilitation. Fever was noted on day 146, and broad-spectrum antibiotic therapy was not effective. Further investigation revealed positive serum cytomegalovirus antigen levels. Administration of ganciclovir 300 mg/day was initiated, but further complication of melena was noted. Gastroscopy was performed and showed cytomegalovirus esophagitis (Fig. 7). The patient died 6 months later (204 days) after hospitalization due to the progression of uncontrollable infection.

## Discussion

The prevalence of malignancy in patients with dermatomyositis is estimated at approximately 20–30% [28]. An anti-TIF1γ antibody associated with malignancy has been identified in dermatomyositis [7]. This antibody is confirmed in approximately 20% of adult patients with dermatomyositis, and 60% to 90% of these patients have malignant disease [6, 28]. The treatment order for cancer-associated DM has not been established, especially whether internal treatment or surgical resection should occur first. To our knowledge, this is the first reported case of anti-TIF1γ antibody-positive dermatomyositis associated with colon cancer.

The major clinical features of the 21 previously reported cases of anti-TIF1γ antibody-positive dermatomyositis associated with cancer and our case are summarized in Table 1 [7–27]. We also investigated the relationship between outcome and treatment, since we faced difficulties in determining the treatment priority between surgical resection and internal treatment. The mean age (± standard deviation) of the population was 63.7 ± 13.7 years (range, 22–83 years), of whom 12 were male and 10 were female. The most common presenting symptom was rash (86%), followed by muscle weakness (50%), dysphagia (45%), facial edema (14%), and myalgia (14%). Although all cases were associated with concurrent DM and cancer, initial diagnosis of DM (68%) was more prevalent than that of cancer (32%). Most of the cases were single cancer; however, two cases of double cancer and one case of triple cancer were noted. The types of cancer included lung (*n* = 7), breast (*n* = 5), gastric (*n* = 5), colon (*n* = 1), esophageal (*n* = 1), urothelial (*n* = 1), pancreatic (*n* = 1), thyroid (*n* = 1), thymic (*n* = 1), ovarian (*n* = 1 case), extragonadal germ cell tumor (*n* = 1), and myelodysplastic syndrome (*n* = 1). Creatine kinase values differed widely. Our case reported the maximum value of creatine kinase (15,667 U/L) among all cases in the literature. The option for treatment showed surgical treatment to internal treatment in five cases, whereas the opposite was true in seven cases. Internal treatment only was performed in nine cases, and surgical treatment only was performed in one case. Regarding response to internal treatment, “partial response” was defined as temporary remission of the symptoms and creatine kinase level only to worsen later. “No response” was defined as progressive symptoms over time. Remission was noted in five cases and partial response was observed in eight cases, while seven cases showed no response to the internal treatment.

**Table 1:** The major clinical features of the 21 previously reported cases of anti-TIF1γ antibody-positive dermatomyositis associated with cancer and our case

Case	Reference/Year	Author	Age (years)	Sex	Chief complaint	Initial diagnosis	Cancer type	Initial CK (U/L) (Maximum value if noted)	Order for treatment	Treatment	Response to internal treatment	Outcome from the diagnosis of DM
1	[7], 2013	Ito	59	M	Rash	DM	Gastric cancer IgG4-positive pulmonary inflammatory pseudotumor	97	Surgical to internal treatment	Surgical resection (stomach resection and right lower lobectomy) Prednisolone	Remission	Alive but no detailed described
2	[8], 2016	Ogawa	63	F	Facial edema	Cancer	Breast cancer	2326	Surgical to internal treatment	Surgical resection (partial excision of left breast) Chemoradiotherapy Tacrolimus	ND	Alive at 11 months
3	[9], 2016	Kubecek	43	M	Fever Fatigue Myalgia Dysphagia Rash	DM	Breast cancer	1574	Internal treatment	Steroid pulse Prednisolone Chemoradiotherapy	No response	ND
4	[10], 2016	Taki	22	M	Rash	DM	Extragonadal germ cell tumor	Normal	Internal to surgical treatment	Prednisolone Chemoradiotherapy Surgical resection (tumor resection, left orchiectomy and retroperitoneal lymph node dissection)	Partial response	Alive at 7 months
5	[11], 2016	Murase	73	M	Rash Muscle weakness Dysphagia	DM	Gastric cancer	1266	Internal treatment	Prednisolone chemotherapy	No response	Dead after 95 days
6	[12], 2017	Matsushita	66	F	Dysphagia Muscle weakness Rash	Cancer	Breast cancer	864	Internal to surgical treatment	Prednisolone	No response	Dead after few months
7	[13], 2017	Palterer	78	F	Muscle weakness Dysphagia Rash Facial edema	DM	Myelodysplastic syndrome	56	Internal treatment	Steroid pulse IVIG Methotrexate Prednisolone	Partial response	Dead after 1 year
8	[14], 2017	Kikuchi	69	F	Rash	DM	Papillary thyroid cancer Breast cancer Gastric cancer	536	Internal to surgical treatment	Prednisolone Surgical resection (Thyroid gland, cervical lymph node, left breast, total stomach and gallbladder resection)	Partial response	Dead after 18 months
9	[15], 2017	Schiffmann	64	F	Rash	Cancer	Gastric cancer	Normal	Surgical treatment	Surgical resection (stomach resection)	NA	Alive at least 2 months later
10	[16], 2018	Karino	72	M	Rash	DM	Thymic carcinoma	1576	Surgical to internal treatment	Surgical resection (thymus resection) Prednisolone	Remission	Alive at least 1 year later
11	[17], 2018	Teraishi	42	F	Rash Muscle weakness	DM	Breast cancer (1st) Ovarian cancer (2nd)	500 (1st) 232 (2nd)	Surgical to internal treatment (1st) Internal to surgical treatment (2nd)	Surgical resection (breast resection) Chemotherapy Prednisolone (1st) Chemotherapy Surgical resection (right salpingo-oophorectomy, omentectomy, and pelvic lymph node dissection) Prednisolone (2nd)	Remission (1st) Remission (2nd)	Alive at 8 years (1st) Alive at least 5 months later (2nd)
12	[18], 2019	Aritomi	63	F	Cough Hoarseness	Cancer	Small cell lung cancer	3272	Internal treatment	Chemotherapy Prednisolone	No response	Dead after a few months
13	[19], 2019	Kato	68	F	Rash	DM	Small cell lung cancer	252	Internal to surgical treatment	Chemotherapy	Partial response	ND
14	[20], 2019	Saraya	58	M	Rash Cough Muscle weakness Dysphagia	DM	Lung adenocarcinoma	7833	Internal treatment	Prednisolone Chemoradiotherapy	No response	Dead after 6 months
15	[21], 2019	Varedi	65	F	Myalgia Dysphagia Rash	DM	Pancreatic neuroendocrine tumor	Normal	Internal treatment	Mycophenolate mofetil Hydroxychloroquine Prednisolone Methotrexate Tacrolimus ointment Surgical resection (pancreas resection)	Remission	Alive at 2 months
16	[22], 2019	Shibata	71	M	Rash Dysphagia	Cancer	Gastric cancer	300 (>1000)	Internal treatment	Chemotherapy Nivolumab Prednisolone Steroid pulse IVIG Tacrolimus	No response	Dead after 142 days
17	[23], 2020	Zarkavelis	72	M	Muscle weakness Rash	Cancer	Urothelial carcinoma	1025	Surgical to internal treatment	Surgical resection (cystoprostatectomy and right ureteronephrectomy) Ipilimumab Nivolumab Prednisolone IVIG	Remission	Alive at least 5 months later
18	[24], 2020	Nakanishi	80	F	Dyspnea Dysphagia Muscle weakness	DM	Lymphoepithelioma-like carcinoma	268	Internal treatment	Steroid pulse Prednisolone Radiotherapy	Partial response	Alive at least 6 months later
19	[25], 2020	Kuczmarska-Haas	83	M	Rash Muscle weakness Dysphagia	DM	Small cell lung cancer	ND	Internal treatment	Prednisolone IVIG Radiotherapy	Partial response	Alive at least 6 months later
20	[26], 2020	Osaki	64	M	Rash Muscle weakness	Cancer	Lung adenocarcinoma	6381	Internal treatment	Nivolumab Chemotherapy Prednisolone IVIG	No response	Dead after 6 months
21	[27], 2020	Sumazaki	70	M	Muscle weakness Myalgia Rash	DM	Esophageal cancer	6727	Internal to surgical treatment	IVIG Surgical resection (esophagectomy with 2-field lymph node dissection) Prednisolone	Partial response	Dead after 3 year
22	2020	Ono	57	M	Facial edema Brachial edema Muscle weakness Dysphagia Myalgia Rash	DM	Ascending colon cancer	5002 (15,667)	Internal to surgical treatment	IVIG Steroid pulse Prednisolone Azathioprine Surgical resection (right hemicolectomy, gastrostomy, and tracheostomy)	Partial response	Dead after 6 months

*CK* creatine kinase, *DM* dermatomyositis, *F* female, *IgG4* immunoglobulin G4, *IVIG* intravenous immunoglobulin, *M* male, *NA* not applicable, *ND* not described

As for treatment for DM, systemic steroid therapy is considered the gold standard. Oral prednisolone at an initial dose of 0.5–1 mg/kg/day followed by a slow progressive dose reduction is recommended. In patients with severe disease, steroid pulse of intravenous mPSL 1000 mg for three consecutive days is also an option for treatment. In addition, the introduction of IVIG or immunosuppressive medications such as methotrexate, azathioprine, cyclophosphamide, or ciclosporin is another option if the patient does not respond to steroid therapy or suffers adverse side effects [9, 29]. Although these internal treatments are essential for the control of DM, the risk of surgical treatment will increase due to the immunocompromised effect. Since tapering of the prednisolone dose takes a relatively long time, the timing of surgery before or after internal treatment is important. In our case, surgical treatment was delayed due to uncontrollable dysphagia and long-term use of steroid therapy. The treatment for cancer in the case reports included surgical resection and chemoradiotherapy. Immune checkpoint inhibitors such as nivolumab and ipilimumab were reported in  three cases [22, 23, 26]. One report noted that only surgical removal of the tumor resulted in the disappearance of the skin rash of DM [15].

The outcomes revealed that half of the patients were alive while the others had died. We further investigated the relationship between outcome and dysphagia, initial treatment, and maximum creatine kinase values. Table 2 demonstrates this relationship. First, dysphagia is a major complication of DM because it leads to oral feeding difficulties and malnutrition [22]. Table 2 (a) shows the relationship between dysphagia and outcome; no significant difference (*P* = 0.12; Pearson’s chi-square test) was noted between them, although the presence of dysphagia tended to be associated with a worse outcome. Table 2 (b) shows the relationship between initial treatment and outcome. Of note, initial surgical treatment led to better outcomes (*P* = 0.0007; Pearson’s chi-square test). However, interpretation must consider potential bias, as patients who are able to undergo surgery may have better general condition. In fact, patients with early-stage disease could firstly undergo surgery according to the cancer stage in Table 1. Conversely, the patients with initial internal treatment include unresectable cancers, in which surgical resection itself is not applicable at the point of diagnosis [9, 13, 20, 22, 24, 26]. Table 2 (c) shows the relationship between the maximum creatine kinase level and outcome. In all four cases where the level was greater than 5000 U/L, the patients died (*P* = 0.033; Pearson’s chi-square test). Lastly, the relationship between response to internal treatment and outcome is shown in Table 2 (d). In five of the cases with remission, the patients were alive, and in the group with no response, six patients had died. Among those with partial response, three patients were alive and four had died. These results suggest that a response to internal treatment is needed for lifesaving results.

**Table 2:** The relationship between outcomes and the presence of dysphagia (a), the type of initial treatment (b), maximum creatine kinase level (c), and response to internal treatment (d)

(a)			
	Dysphagia		
	(+)	(−)	
Alive	3	7	
Dead	7	4	
(b)			
	Initial treatment		
	Surgical	Internal	
Alive	6	4	
Dead	0	10	
(c)			
	Max creatine kinase (U/L)		
	<5000	>5000	
Alive	9	0	
Dead	6	4	
(d)			
	Response to internal treatment		
	Remission	Partial	No response
Alive	5	3	0
Dead	0	4	6

## Conclusions

In conclusion, we reviewed and summarized previously reported cases of anti-TIF1γ antibody-positive DM with malignancy. Cancer screening is essential in patients with anti-TIF1γ antibody-positive dermatomyositis because it is associated with a high prevalence of malignancies. Our review revealed that initial surgical treatment should be recommended for better prognosis if the general condition allows.

## Data Availability

Not applicable.

## Abbreviations

DM: Dermatomyositis
IVIG: Intravenous immunoglobulin
TIF1γ: Transcriptional intermediary factor 1 gamma
mPSL: Methylprednisolone
MRI: Magnetic resonance imaging
